# To see, or not to see… pathogens in virtual reality hand hygiene training

**DOI:** 10.1017/ice.2024.135

**Published:** 2024-10

**Authors:** Aline Wolfensberger, Juliette C. Désiron, Beatrice Domenech-Jakob, Dominik Petko, Walter Zingg

**Affiliations:** 1 Department for Infectious Diseases and Hospital Epidemiology, University Hospital of Zurich, University of Zurich, Zurich, Switzerland; 2 Institute for Implementation Science in Health Care, University of Zurich, Zurich, Switzerland; 3 Institute of Education, University of Zurich, Zurich, Switzerland

## Abstract

**Background:**

ViRTUE, a virtual reality (VR) hand hygiene trainer, offers users the option of visualizing pathogen transfers during virtual patient care either in “real-time” or at the end of a level as a “summary” visualization. In this study, we aimed to evaluate the effect of different timings of pathogen visualization (“real-time” vs “summary”) on in-trainer performance and user’s immersion.

**Methods:**

The study included first-year medical students undergoing hand hygiene training with ViRTUE, randomized to one of three visualization set-ups: set-up 1 (“on-off-off”, with “real-time” visualization at the first level only, and “summary” visualization at level 2 and 3), set-up 2 (“off-on-off”), and set-up 3 (“off-off-off”). In-trainer performance was defined by number of pathogen transmission events (=contaminations) in level 3. The virtual experience of user’s (among others: immersion) was assessed with a questionnaire.

**Results:**

173 medical students participated in the study, with 58, 54, and 61 assigned to set-up 1, set-up 2, and set-up 3, respectively. Users assigned to set-up 3 with “summary” visualization at all levels, performed best with 1.02 (standard deviation (SD) +/- 1.86) contaminations, compared to 2.34 (SD +/- 3.09) and 2.07 (SD +/- 2.52) contaminations of users assigned to the other set-ups. “Summary” visualization at all levels also resulted in higher immersion of users.

**Conclusions:**

“Real-time” visualization of pathogen transmission during VR hand hygiene training with ViRTUE may negatively affect in-trainer performance and user immersion. This emphasizes the importance of pilot testing the effect of VR-based trainings in order to understand their impact on users.

## Background

Transmission of multidrug-resistant organisms (MDROs) are serious adverse events for hospitalized patients, particularly when resulting in healthcare-associated infections (HAIs). Hand hygiene performed by health care workers (HCW) is a well-recognized measure effectively reducing MDRO-transmission and HAI. Training and education on hand hygiene indications and technique have become standard procedures, but implementation is challenging.^1^ The World Health Organization’s (WHO) “Guidelines on Core components of Infection Prevention and Control (IPC) Programmes” advises that “*IPC education should be in place for all HCW […] and include bedside and simulation training”*.^2^

Simulation training is well recognized to educate HCWs, by allowing them exposure to near real-life situations in which they can practice repeatedly in a safe context. Simulation training using immersive virtual reality (VR) allows users to immerse themselves in a virtual environment with a first-person perspective.^3^ A recent systematic literature review on VR in education identified a number of applications of VR in the medical field and reported that a majority of researchers used this technology to increase intrinsic motivation of students.^4^ In addition, VR is used to immerse learners in situations that would be difficult to train by other means. This allows for experiential learning in domains where training would be difficult or even dangerous.

A major challenge in HAI-prevention is the fact that infections become clinically apparent with considerable delay to the causing event, and that microorganisms are only visible with the help of enlarging devices, such as a microscope. Thus, HCWs do not receive immediate feedback about transmission events they caused by non-adherence to hand hygiene moments. Here, VR adds value to simulation training because technology allows to make invisible things visible. ViRTUE, a VR trainer for hand hygiene, is an interactive tool in which users learn hand hygiene behavior experientially.^5^ The “pathogen-view mode” of the trainer allows visualization of microorganisms; thus, allowing users to see otherwise invisible microorganisms. The “pathogen-view mode” is a unique feature of ViRTUE, but its effectiveness and the ideal mode of its use were not yet investigated. With this study, we aimed to determine the optimal moment for visualizing microorganisms – “real-time” versus “summary” - to enhance in-trainer performance.

## Methods

### Virtual reality trainer

**Figure 1.**
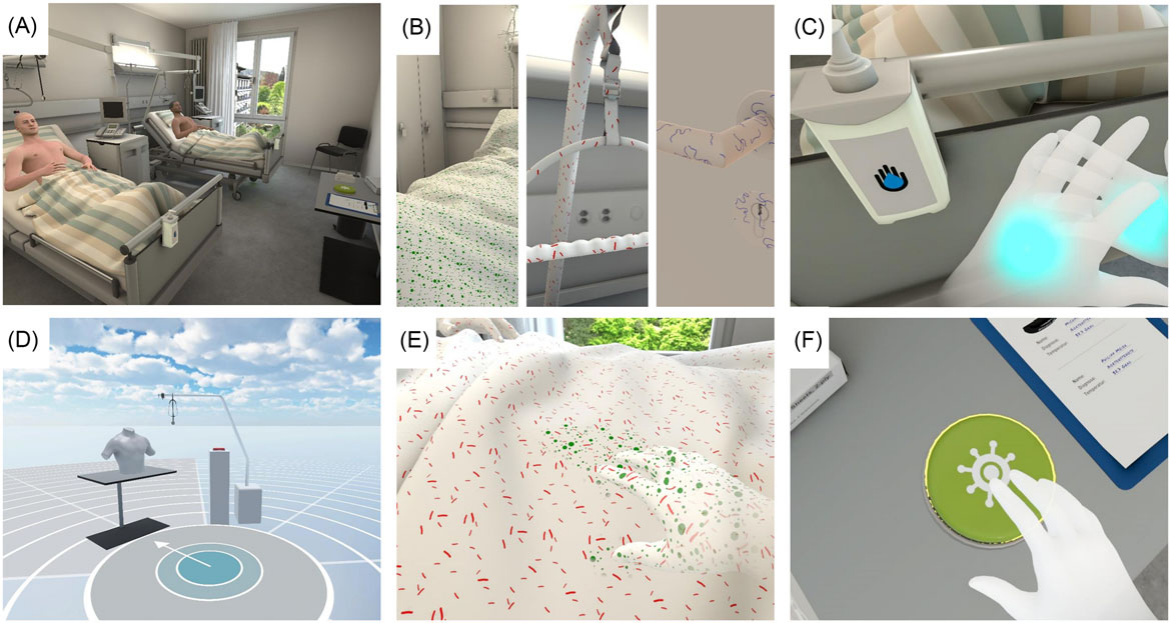
Screenshots of the ViRTUE trainer. A) Virtual patient room accommodating two patients; B) three different categories of microorganisms: green and round for patient/patient zone 1, red and rod-shaped for patient/patient zone 2, purple and spiral for hospital environment; C) hand rub dispenser and moment of performing hand hygiene; D) tutorial environment to learn handling items (stethoscope, ear thermometer, hand disinfection); E) “real-time” visualization of pathogen transmission, here contamination of bed sheet of patient 2 with microorganisms of patient/patient zone 1; F) virtual button for users to switch on “summary” visualization.

ViRTUE uses a virtual patient room accommodating two patients (Figure 1a),^5^ in three levels with increasing difficulty. In all levels, users repetitively complete the same series of four tasks (shaking the hand of the patient, auscultating the patient’s heart, taking the body temperature with an ear thermometer, and writing in the patient chart) for both patients. Three categories of microorganisms are visualized: pathogens of the first patient and his patient zone (green and round); pathogens of the second patient and his patient zone (red and rod-shaped); and pathogens of the hospital environment (purple and spiral) (Figure 1b). Three virtual hand disinfection dispensers are available, one on the wall by the patient room door, and one each at both foot rails of the patient beds. Use of hand rub dispensers removes pathogens from the hands of the user (Figure 1c).

The use of ViRTUE is supervised and assisted by an instructor. Before the first level, users undergo a short tutorial to learn handling items such as the stethoscope, the ear thermometer, and hand disinfection (Figure 1d). At level 1, users are invited by the instructor to discover the surroundings, trigger and watch transmission as a function of their actions (real-time or at the end of the level). The goal in level 2 and 3 is to avoid the transfer of microorganisms, in the level 3 the tasks have to be completed in 180 seconds (timer displayed on the wall). The goal is met if 1) no patient *colonization* happens, ie, by transmission of pathogens to a patient, either from the other patient (or his patient zone) or from the hospital environment and 2) no *contamination* happens, ie, by transmission of pathogens to a patient, a patient zone or the hospital environment (Figure 1e). The software settings allow for a maximum of two colonizations (maximum one per patient) and an infinite number of contaminations.

The “pathogen-view mode” of ViRTUE makes microorganisms visible. The user can choose to see the pathogens in “real-time” visualization during a level, ie, while carrying out the patient care tasks, or as a “summary” visualization at the end of the level by pushing a virtual button (Figure 1f). In addition to visualizing the microorganisms, the “real-time” visualization includes prompting a “ding” sound at each contamination, and the colonized patient starts coughing.

### Participants and data collection

Study participants were first-year medical students at the University of Zurich, participating at a mandatory course on hand hygiene and infection prevention and control (IPC). The students did not attend any lecture about infection prevention during their curriculum until this time, nor was a theoretical instruction on hand hygiene indications included in the VR trainer. In addition to ViRTUE, the course included two workstations on hand hygiene indications and hand hygiene technique. All students were invited to participate in the study; only data from students who gave informed consent were included. No monetary compensation or course credit was offered in exchange of study participation. Based on G*Power a-priori analysis for analyses of covariance *f* = 0.30, *α* = 0.05, power = 0.95, we aimed for a minimum sample of 175 participants.

### Intervention

To investigate the best moment for visualizing microorganisms, we configured three different set-ups. In set-up 1 (“on-off-off”), the “real-time” pathogen visualization was activated at level 1, and deactivated at levels 2 and 3, ie, meaning that “summary” visualization was provided at the end of levels 2 and 3. In set-up 2 (“off-on-off”), visualization was “real-time” at level 2, and “summary” at level 1 and 3. In set-up 3 (“off-off-off”), there was no “real-time” visualization at all levels. Users were randomly assigned to one of the three experimental set-ups. Virtual training lasted a maximum of 20 minutes. At the end, students were asked to complete a questionnaire on their mobile phone by scanning a QR code. The questionnaire assessed demographics (age, gender, education degree, and player profile), virtual experience, and technology acceptance (Appendix 1).

### Outcomes

#### In-trainer performance

Three performance indicators were measured at all levels: 1) the number of patient *colonizations*, 2) the number of *contaminations*, 3) time to complete the level (*duration*). Performance at level 3 was the main outcome. This performance data were recorded within the ViRTUE software for each participant.

#### Virtual experience

Three self-reported indicators measured virtual experience in the post-test questionnaire: *immersion* (sense of being there), *representational fidelity* (realism of the environment), and *user engagement* (quality of user experience). *Immersion* and *representational fidelity*, derived from the Cognitive Affective Model of Immersive Learning,^6^ were assessed with questions from the igroup presence questionnaire,^7^ with the four positively framed items of “general and special presence” corresponding to *immersion,* and the four items for “experienced reality” corresponding to *representational fidelity* (answer scales ranging from 1-*strongly disagree* to 5-*strongly agree*). *User engagement* was assessed by an overall score of the 12-items scale by O’Brien et al. (answer scales ranging from 1-*strongly disagree* to 5-*strongly agree*) (full questionnaire see Appendix 1).^8^

#### Technology acceptance

As previous research reported that technology acceptance, particularly behavioral intention and effort expectancy, could affect the performance within a virtual environment,^9^ we assessed the core components of the original technology acceptance model,^10^ ie *performance expectancy* (belief that using a technology may help improve ones’ performance), *effort expectancy* (belief that a technology will be easy to use), and *behavioral intention* (intent towards use of a technology) by questions adapted from the scale developed and validated by Shen et al.^11^ in the post-test questionnaire.

### Statistical analysis

The set-ups were tested by multivariate analysis of covariance (MANCOVA) at level 3: *colonizations*, *contaminations,* and *time*. Set-ups were modeled as independent variables, in-trainer performance indicators as dependent variables. Reliability analysis was performed on the six measures for virtual experience and technology acceptance by using Cronbach α and McDonald’s ω. Based on Pearson’s correlation analysis between virtual experience items, technology acceptance items and in-trainer performance indicators, variables of virtual experience and technology acceptance significantly correlating with performance indicators were modeled as covariates (Appendix 2).

To compare self-reported virtual experience between set-ups, we conducted a multivariate analysis of covariance (MANCOVA), with *immersion*, *representational fidelity,* and *engagement* as dependent variables, and effort expectancy and performance expectancy as covariates. The statistical analyses were conducted with the statistical software jamovi, version 2.3.^12^

## Results

Of 177 medical students attending the course, 173 provided informed consent and were included in the study. The majority (64.6%) of participants were female. The mean age was 20 years and 4 months (standard deviation (SD): 2 years and 3 months; *n*_missing_ = 12). Most participants had a high school degree (92.5%), the remainder a bachelor’s or master’s degree. Half (51.5%) considered themselves to be novice video game players, and about a third (28.6%) to be casual players, while the others reported as intermediate (16.1%) to expert players (3.7%). Six participants had played VR video games before.

### In-trainer performance

A total of 58, 54, and 61 students were randomized to set-up 1 (“on-off-off”), set-up 2 (“off-on-off”), and set-up 3 (“off-off-off”), respectively. At level 3, a mean of 0.09 (SD +/- 0.28), 0.06 (SD +/- 0.22), and 0.0 (SD +/- 0.0) patients were colonized in set-up 1, set-up 2, and set-up 3, respectively. A mean of 2.34 (SD +/- 3.09), 2.07 (SD +/- 2.52), and 1.02 (SD +/- 1.86) contaminations happened, in set-up 1, set-up 2, and set-up 3, respectively. Participants passed level 3 at a mean of 120 (SD +/- 26.4), 126 (SD +/- 23.0), and 130 (SD +/- 25.1) seconds in set-up 1, set-up 2, and set-up 3, respectively. Table 1 summarizes colonizations and contaminations by level and set-up.

**Table 1.** Colonization and contamination scores per level and set-up

	Colonization						Contamination					
	Level 1		Level 2		Level 3		Level 1		Level 2		Level 3	
	M	±SD	M	±SD	M	±SD	M	±SD	M	±SD	M	±SD
Set-up 1 “on-off-off”	1.88	0.46	0.21	0.45	0.09	0.28	10.40	6.41	2.24	3.32	2.34	3.09
Set-up 2 “off-on-off”	1.37	0.78	0.06	0.23	0.06	0.22	8.87	7.22	1.69	1.82	2.07	2.52
Set-up 3 “off-off-off”	1.90	0.30	0.07	0.25	0.0	0.0	11.50	5.52	1.93	2.26	1.02	1.86

**Caption**: Mean scores (+/- SD) of colonizations and contaminations per set-up and level. “On” signifies that visualization of pathogens was in “real-time,” while “off” signifies that the visualization of pathogens was provide as “summary” at the end of each level.

**Abbreviations**: M, mean; SD, standard deviation.

After correcting for co-variates, there was a significant main effect of “set-up” on in-trainer performance (Wilk’s Λ = .91, *F* (6,308) = 2.48, *P* = .024). Follow-up univariate analyses were significant for the number of contaminations, *F* (2,155) = 3.84, *P* =.024, *η* ^2^_p_ = 0.047, but neither for the number of colonizations (*P* = .119) nor for duration (*P* = .306). Post hoc comparison using Tukey correction showed that participants in set-up 3 (“off-off-off”) caused significantly less contaminations compared to participants in set-up 1 (“on-off-off”) (*P* = .023).

### Virtual experience and technology acceptance

The results of virtual experience and technology assessment are summarized in Table 2. *Immersion* (ie the sense of being there) reached the highest values with means of the Likert scale between 4.09 (SD 0.55) and 4.33 (SD 0.45). *Representational fidelity* (realism of the environment) and *user engagement* (quality of user experience) reached means between 3.12 and 3.37 (of a maximum of 5). High scores were obtained in *performance expectancy* (ie that technology helps to improve behavior) and *effort expectancy* (ie, that technology will be easy to use) (5.31 to 5.75); however, *behavioral intention* (ie the intent to use the technology) scored lower (4.72 to 4.94). Reliability analyses for all measures showed overall moderate to good results.

**Table 2.** Virtual experience and technology acceptance score per set-up

	Virtual experience						Technology acceptance					
	Immersion (max. 5)		Representational fidelity (max. 5)		User engagement (max. 5)		Performance expectancy (max. 7)		Effort xpectancy (max. 7)		Behavioral intention (max. 7)	
	M	±SD	M	±SD	M	±SD	M	±SD	M	±SD	M	±SD
Set-up 1 “on-off-off”	4.16	0.50	3.30	0.53	3.37	0.33	5.58	1.07	5.68	0.856	4.94	1.32
Set-up 2 “off-on-off”	4.09	0.55	3.12	0.56	3.33	0.33	5.31	1.26	5.75	0.875	4.72	1.24
Set-up 3 “off-off-off”	4.33	0.45	3.30	0.51	3.31	0.30	5.32	0.91	5.64	0.837	4.88	1.05

**Caption**: Mean scores (+/- SD) per set-up and item of “virtual experience” and “technology acceptance”. “On” signifies that visualization of pathogens was in “real-time,” while “off” signifies that the visualization of pathogens was provide as “summary” at the end of each level.

**Abbreviations**: M, mean; SD, standard deviation.

There was an overall significant effect of set-up on virtual experience (Wilk’s Λ = .91, *F* (6,308) = 2.44, *P*=.026). Follow up univariate analyses further showed that the effect was significant for *immersion* (*F* (2,154) = 4.42, *P* =.014, η2p = 0.054), but neither for *representational fidelity* (*p* = .093) nor for *engagement* (*P* = .456). Post hoc comparisons with Tukey correction revealed that differences between set-up 1 and set-up 2 were not significant (*P* = .975), but that differences in immersion between set-up 1 and set-up 3 (*P* = .042), or set-up 2 and set-up 3 (*P* = .024) were reaching significance.

## Discussion

ViRTUE is a hand hygiene trainer allowing visualization of microorganism transfer in a virtual patient room. Users can choose between “real-time” visualization during a level or a ‘summary’ visualization at the end of the levels. In this study with users assigned to a specific set-up, we found that users who were assigned to “summary” visualization in all levels performed best and that immersion (the “sense of being there”) was highest for that set-up as well.

In medical education, VR applications have been used for teaching in many medical fields such as anatomy,^13^ endoscopy,^14^ vascular access,^15^ or transvenous lead extraction of implantable cardiac devices.^16^ The results of recent systematic reviews suggests that VR-based education results in better pass rates compared to conventional education modes,^17^ and that VR results in higher postintervention knowledge and better skills compared to other digital education modes.^18^ Studies evaluating the effectiveness of VR-based hand hygiene training compared to traditional education show conflicting results; some studies showed no difference,^19,20^ while others supported the use of VR-based education.^3^ The effectiveness of ViRTUE to improve adherence to the hand hygiene moments as compared to traditional modes of education had not been yet assessed, but in a previous study, medical students using ViRTUE were found to perform significantly better at level 3 compared to level 1.^21^

The concepts of available immersive VR-based hand hygiene trainers differ. “VR clean hands” published by Eichel et al., includes a practical task training on indication and sequence of hand hygiene in a variety of practical clinical situations within three patient rooms.^19^ Feedback on errors is given to the participant as an immediate warning. Omori et al. published about a fully immersive 360-degree video that was filmed during a patient examination and displays the experience both from the doctors’ and the patients’ point of view.^3^ The spread of methicillin-resistant *Staphylococcus aureus* (MRSA) to environment is visualized using computer graphics. Both VR trainers include a theoretical description of correct hand hygiene.^3,19^ ViRTUE is conceptually different from those two trainers, as it visualizes transmission of microorganism caused by the user. Seeing one’s own transmission events allows experiential learning,^22^ a learning approach that was shown to result in better learning outcomes and promoting a more positive experience compared to conventional learning.^23^

Little is known about how to best train medical students on hand hygiene in general, and even less is known about utilizing technical support such as the virtual space. In a previous study, we assessed how users are best prompted (ie, instructed about how to act in the virtual space) and found that asking them to deliberately do mistakes did not increase performance.^24^ In the present study, we found that the immediate visual feedback of pathogen transmission by “real-time” pathogen visualization during performing tasks – compared to “summary” visualization at the end of each level – does not have a positive (but potentially a negative) effect on the performance. The reason for this finding remains speculative. It might be that the users who have taken advantage of observing the immediate effects of non-adherence might not have had sufficient time to practice high adherence, while the users in the “off-off-off” set-up might have benefitted from the lack of variation between the levels. As the immediate visibility of pathogen transmission can be inviting to actively doing mistakes, another potential reason could be that the users were distracted or had difficulties to “switch-back” from the experiential mode of observing the consequences of doing mistakes to a mode where high adherence was demanded. Possibly, we were observing a similar effect as in the aforementioned study examining two prompts: users who were prompted to “explore and do mistakes” sticked to doing mistakes in the next level, compared to users that were prompted to “do your best”.^24^ Last, the higher feeling of immersion of the group that was only practicing in real-time pathogen-view mode off (“off-off-off”) might have led to higher sense of responsibility for the (virtual) patients’ health and thus higher adherence.

Our study has some limitations: first, the tasks the users must carry out during the three training levels are not overly complex but are designed for novice medical students. Consequently, the quantification of set-up effect may be hindered due to restrictions on the number of possible contamination events and patient colorizations. Second, we did not assess the feeling of nausea or dizziness in our participants, a side effect that is well known from the use of VR applications.^19^ Third, the most interesting and relevant outcome measure – hand hygiene performance in the real world – was not assessed in this study. Future research would be needed to investigate the real-life effectiveness of ViRTUE, also in comparison to non-virtual or other media training. Fourth, the findings of our study are limited to the study population of first-year medical students and the very specific design of the ViRTUE trainer. And last, our study was slightly underpowered (n = 173 instead of 175 participants) which might limit the reliability and validity of our results.

This study sheds some light on how VR hand hygiene trainers like ViRTUE could be designed to teach students in the best possible way. Even though ViRTUE offers the possibility to observing transmission events in real-time, this does not yield improved hand-hygiene in-trainer performance in novice medical students but on the contrary might decrease short-term performance. Further exploration is required to uncover the underlying reasons, for example through the analysis of qualitative interview data from users. Moreover, assessing post-ViRTUE hand hygiene compliance in real-life is imperative for gauging the tool’s long-term effectiveness.

## Supporting information

Wolfensberger et al. supplementary material Wolfensberger et al. supplementary material

## Supplementary material

The supplementary material for this article can be found at https://doi.org/10.1017/ice.2024.135
