# Supplementary material for: To see, or not to see… pathogens in virtual reality hand hygiene training
Source: Infect Control Hosp Epidemiol. 2024 Oct 15;45(10):1202–7. doi: 10.1017/ice.2024.135 (PMC11611501; doi:10.1017/ice.2024.135)
Supplement: Wolfensberger et al. supplementary material [file S0899823X24001351sup001.docx]

**Appendix «To see, or not to see… pathogens in virtual reality hand hygiene training»**

# Appendix 1 Questionnaire for assessing virtual experience and technology acceptance

## Virtual experience:

**Immersion:**

Q 1. In the VR trainer I had a sense of «being there».

Q 2. Somehow, I felt that the virtual environment surrounded me.

Q 3. I had the sense of acting in the virtual space, rather than operating something from outside.

Q 4. I felt present in the VR trainer.

**Representational fidelity:**

Q 1. The virtual environment seemed real to me.

Q 2. My experience in the virtual environment was consistent with a real world experience.

Q 3. The VR trainer seemed more realistic than the real world.

Q 4. I felt like I was perceiving more than pictures.

**User engagement:**

Q 1. I lost myself in this experience.

Q 2. The time I spent using the trainer just slipped away.

Q 3. I was absorbed in this experience.

Q 4. I felt frustrated while using this trainer.

Q 5. I found this trainer confusing to use.

Q 6. Using this trainer was demanding

Q 7. This trainer was attractive.

Q 8. This trainer was aesthetically appealing.

Q 9. This trainer appealed to my senses.

Q 10. Using the trainer was worthwhile.

Q 11. My experience was rewarding.

Q 12. I felt interested in this experience.

## Technology acceptance:

**Performance expectancy:**

Q 1. I would find virtual reality useful in my education.

Q 2. Using virtual reality in my education would increase my productivity.

Q 3. Using virtual reality in my education would enhance my effectiveness.

Q 4. Using virtual reality in my education would improve my academic performance.

**Effort expectancy:**

Q 5. My interaction with virtual reality would be clear and understandable.

Q 6. It would be easy for me to become skilful at using virtual reality.

Q 7. I would find virtual reality easy to use.

Q 8. Learning to operate virtual reality would be easy for me.

**Behavioural intentions:**

Q 9. I would like to use virtual reality for learning in the near future.

Q 10. I predict I would use virtual reality in the near future.

Q 11. I plan to use virtual reality for learning in the near future.

# Appendix 2 Correlations (Pearson’s r) between outcome measures of preformance and virtual experience, and with TAM components

|  |  | 1 | 2 | 3 | 4 | 5 |
| --- | --- | --- | --- | --- | --- | --- |
| 1. Contaminations | *r* | — |  |  |  |  |
|  | *p* | — |  |  |  |  |
| 2. Colonisations | *r* | 0.424 | — |  |  |  |
|  | *p* | < .001 | — |  |  |  |
| 3. Duration | *r* | 0.238 | 0.061 | — |  |  |
|  | *p* | 0.002 | 0.426 | — |  |  |
| 4. Immersion | *r* | -0.21 | -0.155 | -0.001 | — |  |
|  | *p* | 0.006 | 0.042 | 0.99 | — |  |
| 5. Representational fidelity | *r* | -0.149 | -0.036 | 0.02 | 0.452 | — |
|  | *p* | 0.05 | 0.64 | 0.792 | < .001 | — |
| 6. Engagement | *r* | -0.078 | 0.05 | 0.039 | 0.384 | 0.401 |
|  | *p* | 0.314 | 0.518 | 0.618 | < .001 | < .001 |
| 7. Behavioural intention | *r* | 0.057 | 0.051 | 0.063 | 0.073 | 0.144 |
|  | *p* | 0.479 | 0.522 | 0.433 | 0.348 | 0.065 |
| 8. Effort expectancy | *r* | -0.162 | -0.003 | -0.075 | 0.324 | 0.364 |
|  | *p* | 0.041 | 0.973 | 0.342 | < .001 | < .001 |
| 9. Performance expectancy | *r* | -0.117 | 0.083 | -0.026 | 0.313 | 0.479 |
|  | *p* | 0.141 | 0.296 | 0.747 | < .001 | < .001 |
